# Predicting, preventing, and identifying delirium after cardiac surgery

**DOI:** 10.1186/s13741-016-0032-5

**Published:** 2016-04-26

**Authors:** Jason B. O’Neal, Andrew D. Shaw

**Affiliations:** Department of Anesthesiology, Vanderbilt University Medical Center, Nashville, TN USA

**Keywords:** Postoperative delirium, Cardiac surgery, Neuroinflammation

## Abstract

Delirium after cardiac surgery is a major problem. The exact mechanisms behind delirium are not understood. Potential pathways of delirium include neurotransmitter interference, global cognitive disorder, and neuroinflammation. Several predisposing and precipitating risk factors have been identified for postoperative delirium. The development of delirium following cardiac surgery is associated with worse outcomes in the perioperative period. Multiple interventions are being explored for the prevention and treatment of delirium. Studies investigating the potential roles of biomarkers in delirium as well as pharmacological interventions to reduce the incidence and duration of delirium are necessary to mitigate this negative outcome.

## Background

Delirium is a syndrome defined as an acute state of confusion and inattention which may be accompanied by an altered level of consciousness and disorganized thinking (Cole 2004). The American Psychiatric Association’s Diagnostic and Statistical Manual, 5th edition (DSM-V), provides five key components of delirium (Association AP 2013): a disturbance in attention and awareness; the disturbance is acute and develops over a short period of time while fluctuating during the course of the day; a disturbance in cognition occurs; these disturbances are not explained by another neurocognitive disorder and do not occur during a state of reduced level of arousal including coma; and there is evidence to suggest that the disturbance is caused by a medical condition, substance intoxication or withdrawal, or side effect of a medication. Other features of delirium may include psychomotor disturbances and variable emotional states. Delirium can be categorized into three subgroups: hypoactive, hyperactive, or mixed. The most common type of delirium is hypoactive. When making the diagnosis of delirium, agitation must not be misinterpreted as hyperactive delirium.

Given the range of symptoms and sub-types, the diagnosis of delirium may be difficult and under-recognized by healthcare professionals (Boustani et al. 2010). Several scales are available to assist in making the correct diagnosis. A comparison of methods in the intensive care unit (ICU) found the confusion assessment method for the ICU (CAM-ICU) to be the most valid and reliable delirium assessment tool when compared with the Nursing Delirium Screening Scale (Nu-DESC) and Delirium Detection Score (DDS) (Luetz et al. 2010). Using these tests is time-consuming, especially for providers not formally trained to complete the assessment. The optimal method(s) for assessing postoperative delirium following surgery has yet to reach a generalized consensus although the CAM-ICU is the most commonly used method by clinicians.

Postoperative delirium has been studied in various surgical patient populations. The most extensively studied groups are patients undergoing orthopedic procedures such as total hip arthroplasty or total knee arthroplasty and cardiovascular surgery patients (van Meenen et al. 2014). Delirium is associated with negative hospital outcomes including a tenfold increased risk of death, a fivefold increased risk of nosocomial complications (Inouye 2006), poor 1-year functional recovery, and postoperative cognitive decline (Saczynski et al. 2012). The long-term cognitive decline seen in some patients after the development of delirium is similar to that of Alzheimer’s patients (Pandharipande et al. 2013). With reports of up to 50 % of patients over 60 years old developing postoperative delirium following cardiac surgery (Rudolph et al. 2009), this patient population poses a major burden for healthcare.

This review article discusses the pathophysiology, risk factors, and complications of postoperative delirium following cardiac surgery. Pharmacological interventions which may reduce postoperative delirium and the potential role of biomarkers in this patient population are presented. In addition, we discuss several considerations for future studies in the field.

## Review

### Pathophysiology

**Fig. 1:**
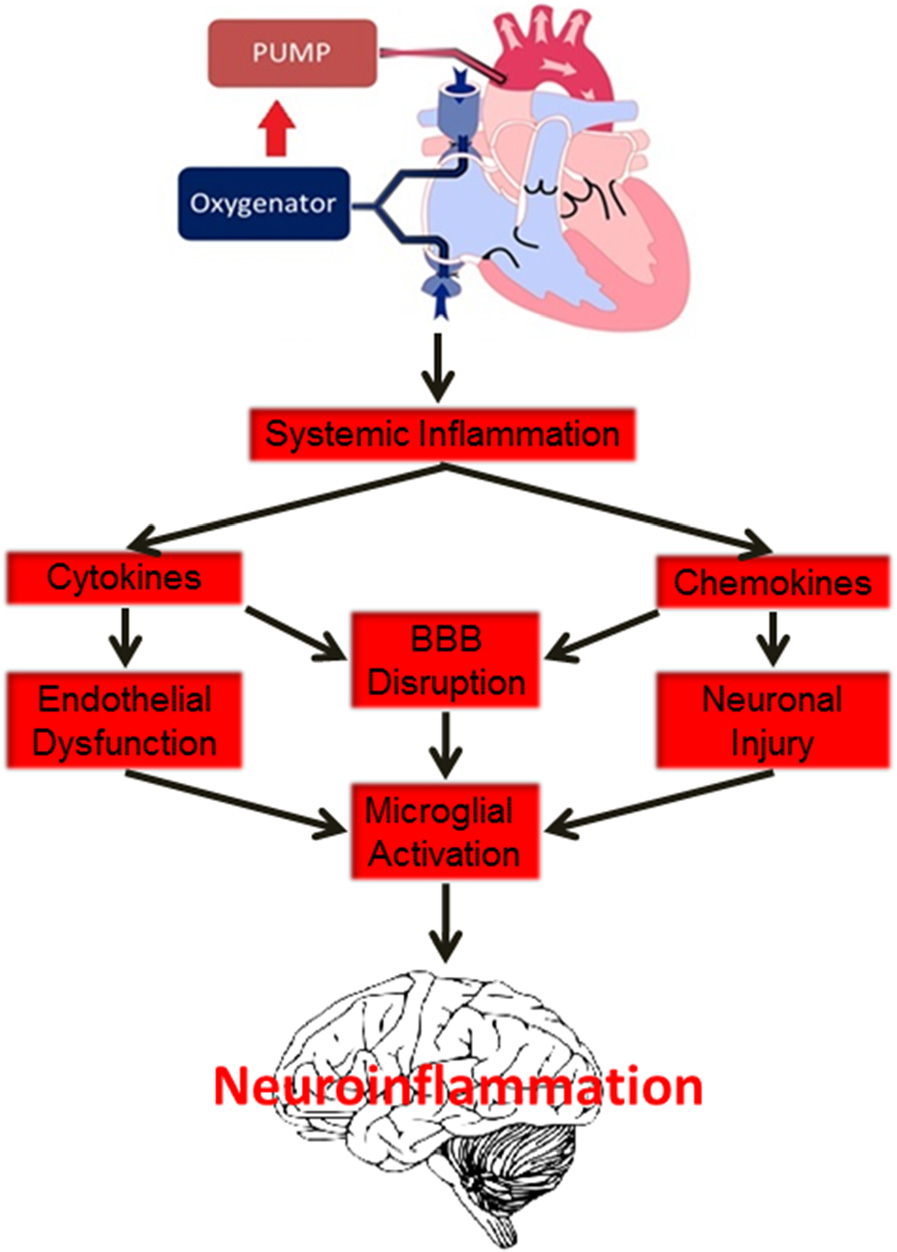
Cardiopulmonary bypass and neuroinflammation

The underlying mechanisms behind delirium are not fully understood. Several hypotheses exist for the pathophysiology of delirium such as neuroinflammation (Fig. 1). The stress associated with cardiac surgery, especially when cardiopulmonary bypass (CPB) is utilized, leads to a systemic inflammatory response. Elevated levels of chemokines, cytokines, and other inflammatory markers may contribute to endothelial dysfunction and disruption of the blood brain barrier (BBB) (Rudolph et al. 2008). When this occurs, the brain is susceptible to neuronal injury via neuroinflammation and the activation of microglia ensues which may be a key component to the development of delirium (Cerejeira et al. 2010).

Neurotransmitter interference may lead to delirium. Reduced levels of plasma cholinesterase activity have a correlation with delirium (Cerejeira et al. 2012; Trzepacz 1996), while increased levels of dopamine may also play a part (Gaudreau & Gagnon 2005). This theory is further supported by evidence which suggests anticholinergic medications and dopaminergic medications instigate delirium (Gaudreau & Gagnon 2005; Trzepacz & van der Mast 2002).

Other theories focus on a more global cognitive disorder in which a single molecule cannot be implicated in delirium, but rather communication in the brain as a whole is impaired. This may include disturbances in the prefrontal and parietal networks, or subcortical structures including the basal ganglia, reticular activating system, thalamic nuclei, and cerebellum (Trzepacz & van der Mast 2002).

In cardiac surgery, the exposure to CPB and the stress endured by the patient produces significant inflammation, and the processes of neuroinflammation provide a logical pathway. This may also hold true in various ICU patients with a diagnosis of sepsis, severe burn injury, or polytrauma. Yet the evidence for an imbalance of certain neurotransmitters and delirium is supported and has been known for years (Cerejeira et al. 2012; Trzepacz 1996). Therefore, delirium is likely multifactorial in nature and components from each theory may be involved.

### Biomarkers

A direction to expand our understanding of the mechanisms behind delirium and to identify patients at increased risk for delirium after cardiac surgery is through targeting biomarkers. Several studies have investigated biomarkers in heart disease, dementia, heart failure, and various settings of delirium, mostly in critically ill patients (Wiltfang 2014; Gaggin & Januzzi 2013; van den Boogaard et al. 2011; Khan et al. 2011). Multiple biomarkers have associations with delirium and may offer support when assessing risk, establishing diagnosis, and quantifying severity of delirium (Khan et al. 2011; Marcantonio et al. 2006).

A focused approach when evaluating biomarkers is necessary, and one that aims to quantify the probable components of neuroinflammation—endothelial dysfunction, BBB compromise, and neuronal injury—as they relate to cardiac surgery and postoperative delirium may be a promising direction. Previous studies have included pro-inflammatory, anti-inflammatory, and also neuron-specific biomarkers. A study investigating both IL-2 and TNF-α in coronary artery bypass grafting (CABG) patients with CPB not only found a correlation between each biomarker and delirium but also provided cutoff levels for increased risk of 907.5 U/ml and 10.95 pg/ml, respectively (Kazmierski et al. 2014). High levels of IL-2 were associated with a higher risk of delirium following cardiac surgery in another study (Baranyi & Rothenhausler 2014). A prospective cohort looking at pterins in postoperative delirium found that the preoperative neopterin level predicted delirium and postoperative neopterin and homovanillic acid levels were associated with delirium after cardiac surgery (Osse et al. 2012). Even with several studies showing associations of specific biomarkers and delirium, a review concluded that not enough evidence existed to support the clinical use of any biomarker for the diagnosis of delirium (Khan et al. 2011). The review did however state that S100B, insulin-like growth factor-1 (IGF-1), and some inflammatory markers demonstrated promising results.

The biomarker S100B has been associated with delirium and acute brain dysfunction in previous studies and is considered a validated measure of BBB disruption (Blyth et al. 2009). BBB disruption and neuron injury via neuroinflammation may contribute to delirium, and therefore, S100B provides support for this theory. Ubiquitin C-terminal hydrolase L1 (UCHL1) is a neuron-specific enzyme recognized in traumatic brain injury patients as a marker for direct neuron injury (Blyth et al. 2011; Brophy et al. 2011). In addition, a link between this enzyme and neurodegeneration has been suggested (Figueiredo-Pereira et al. 2014; Jara et al. 2013). The biomarker UCHL1 has yet to be investigated in delirium and could provide a novel way to predict and screen patients at risk for delirium following cardiac surgery by directly detecting injury to neurons. Another pathway to consider is impaired endothelial vascular reactivity as an association with endothelial vascular injury and brain dysfunction has been demonstrated (Hughes et al. 2013).

### Risk factors

Many risk factors are associated with postoperative delirium. While some may predispose patients to delirium, other risk factors precipitate delirium such as medications given during the perioperative period. Predisposing risk factors are typically long-standing with the patient and should be recognized preoperatively in order to screen patients for increased risk. Precipitating risk factors must be considered by clinicians when treating any patient, especially patients already at high risk for postoperative delirium. Each type of risk factor can be modifiable or non-modifiable, and if possible, those that are modifiable should be optimized in the perioperative period.

Advanced age is a well-recognized risk factor for postoperative delirium. Age has been shown to be an independent risk factor in numerous studies (Chung et al. 2015; Trabold & Metterlein 2014). And with the average age of patients undergoing open-heart surgery in the mid-1960s (O'Neal et al. 2013), many patients automatically fall into a higher risk stratification.

Patients with cognitive impairment are also at risk for postoperative delirium. The incidence of dementia is estimated at 9.8 per 1000 person-years (Ruitenberg et al. 2001), and with humans living longer in a world of steadily increasing population, the prevalence of dementia will continue to rise over the foreseeable future. Impaired cognition may be unrecognized in some patients as people suffering from mild cognitive impairment may present for surgery undiagnosed as symptoms can be subtle at this stage of the disease (Robinson et al. 2012). Thus, these patients are at a greater risk for delirium without healthcare workers being aware. Clinicians should consider screening patients for cognitive impairment prior to surgery and then attempt to avoid and/or mitigate any potential precipitating risk factors in this population.

Other preoperative conditions found to have associations with postoperative delirium include anemia, dehydration, electrolyte abnormalities, and signs of malnutrition (Trabold & Metterlein 2014). A small prospective study of hospitalized patients over the age of 70 years found anemia and dehydration to be risk factors for delirium (Joosten et al. 2006). The finding of anemia as a risk factor was also demonstrated in patients after lumbar spine surgery (Fineberg et al. 2013). This finding has not been reproduced after cardiac surgery; however, whether or not addressing these risk factors prior to surgery can influence the outcome of delirium has yet to be elucidated. Studies which investigate the incidence of delirium after the correction of anemia, dehydration, or other conditions prior to undergoing cardiac surgery should be conducted.

Another consideration is pharmacotherapy before, during, and after surgery. A review of medications taken by patients in the perioperative period of cardiac surgery identified preoperative antipsychotics as an independent risk factor for delirium (Tse et al. 2012). Other preoperative medications that may contribute to delirium include statins, antihypertensives, anticholinergics, antidepressants, benzodiazepines, and opioids which are common home medications for patients undergoing cardiac surgery (Tse et al. 2012). Intraoperative medications associated with delirium include both fentanyl and ketamine while diazepam has inconclusive results (Tse et al. 2012). As for postoperative medications, risperidone was shown to reduce the incidence of delirium by almost one third when taken by patients after cardiac surgery as a preventive strategy (Prakanrattana & Prapaitrakool 2007). However, the large effect size in reducing delirium in patients receiving risperidone (11.1 vs. 31.7 %; RR, 0.35 (95 % CI 0.16 to 0.77); *P* = 0.009) has yet to be reproduced.

Oxygen delivery to the brain is critical for optimal performance of the brain’s activities. Cerebral oximetry continuously measures cerebral oxygenation in both hemispheres of the brain and is sometimes used in cardiac surgery as a monitor to stratify risk in patients throughout the perioperative period (Vretzakis et al. 2014). Previous studies have found a correlation between low preoperative cerebral oxygenation and postoperative delirium (Schoen et al. 2011). Desaturations in cerebral oximetry readings while on bypass are associated with several adverse outcomes (Vohra et al. 2009), but whether or not this may contribute to delirium is unknown.

### Outcomes

Delirium is a condition that may affect a patient for several days or weeks. It is associated with many significant adverse effects and poor outcomes. The progression of dementia after suffering from an episode of delirium may lead to a significant decline in both physical and cognitive functioning (Morandi et al. 2014). This is also evident in patients without an underlying dementia as delirium is associated with declines in both condition and functional level in older patients (Inouye 2006). After cardiac surgery, patients diagnosed with delirium have a higher risk for falls, increased length of stay in the hospital, higher likelihood of discharge to a nursing home or home with assisted care, and prolonged inpatient physical therapy duration (Mangusan et al. 2015). Delirium after CABG surgery is also associated with increased mortality up to 10 years postoperatively (Gottesman et al. 2010).

The outcomes for patients developing delirium are clearly worse than patients who do not. Whether the poor outcomes are a direct effect of delirium or a marker of disease severity is unclear. Either way, these negative outcomes lead to increased costs in not only the healthcare field but also social costs as families incur the increased burdens of time and money to care for relatives diagnosed with delirium. Strategies to reduce healthcare costs are becoming a major focus within the field of medicine. Interventions to effectively treat delirium and its complications are being explored, and strategies to reduce healthcare costs are on the rise (Zaubler et al. 2013). The treatment of delirium is important, but discovering ways to prevent delirium is ideal and would likely equate to the greatest decrease in overall healthcare costs and improved outcomes.

### Pharmacological interventions

The identification and correction of risk factors including dehydration, electrolyte imbalance, and anemia are important and should be emphasized in every patient. And while these non-pharmacological interventions and others such as reorientation, effective communication, and maintenance of consistent sleep-wake cycles are considered first-line interventions for delirium (Young et al. 2010), the discovery of pharmacological agents which decrease the incidence or duration of delirium has great potential. With the current lack of understanding behind the exact pathophysiology of delirium, the present prevention and treatment strategies are inadequate. As previously mentioned, second-generation antipsychotics were shown to decrease the incidence of postoperative delirium when administered prophylactically (Hirota & Kishi 2013); however, only one study in this review was specific to cardiac surgery patients (Prakanrattana & Prapaitrakool 2007). Also, a total of six studies were included in this meta-analysis. Other studies have suggested patients taking preoperative antipsychotics are actually at a greater risk for delirium (Tse et al. 2012). The use of antipsychotics is a potential treatment option as they may alter and subsequently correct neurotransmitter imbalances in delirious patients (Hirota & Kishi 2013; Schotte et al. 1996), but the majority of interventions for delirium after cardiac surgery aim to optimize analgesia and sedation regimens in the ICU and preemptively treat delirium.

Postoperative pain may contribute to the condition of delirium (Fong et al. 2006). Pain after cardiac surgery can be moderate to severe with incisions to the lower extremities and sternum and also the placement of chest tubes. With the potential for bleeding and increased risk of kidney injury, nonsteroidal anti-inflammatory drugs (NSAIDs) are not ideal in this patient population. Ketamine is one option for pain postoperatively, but it may increase the risk of delirium (Tse et al. 2012), and the potential side effect of hallucinations should also be considered. The usual analgesic regimen after cardiac surgery includes an opiate. Opiates, such as morphine and hydromorphone, have many deleterious side effects including nausea/vomiting, ileus, bladder dysfunction, and respiratory depression (Memis et al. 2010). Opioids also increase the risk of postoperative delirium, especially in elderly patients (Tse et al. 2012). The use of intravenous acetaminophen (IVA) has been shown in multiple studies to reduce the amount of opiates consumed by surgical patients (O'Neal 2013). IVA possesses no psychoactive properties, and it has both rapid and effective analgesic properties while considered to be a safe and well-tolerated medication (Candiotti et al. 2010). Given the reduction in opiate consumption, reliable analgesic properties, and safety profile, IVA may be an alternative for patients undergoing cardiac surgery.

In addition to optimizing pain control after cardiac surgery, choosing the most appropriate sedation regimen is important when trying to reduce the chance for postoperative delirium. The importance of periodically weaning sedation should not be overlooked. Overly sedating patients is associated with delayed extubation and higher mortality in the ICU (Shehabi et al. 2013; Shehabi et al. 2012). Finding the appropriate balance of sedation and adequate analgesia should be targeted in cardiac surgery patients.

An ideal choice for sedation in the ICU is a medication that provides both sedation and pain relief. Dexmedetomidine is a selective α-2 receptor agonist which provides sedation and also has an analgesic component. Some clinicians use caution before administering this medication given the side effect profile which includes bradycardia, hypotension, and hypertension with loading doses. Cardiac surgery patients have the advantage of pacer wire implants to avoid the side effect of bradycardia, and since these patients are in the ICU, hypotension can be treated with vasopressors when appropriate. A study comparing sedation with propofol to dexmedetomidine in the ICU found no difference between the two with regard to bradycardia and hypotension; however, dexmedetomidine was associated with an increased risk of hypertension in patients receiving a loading dose initially (Xia et al. 2013). This undesired side effect might be avoided by foregoing the loading dose. Given its sedative and analgesic properties with limited side effects, dexmedetomidine could be the ideal medication for cardiac surgery patients, and multiple studies have investigated the role of dexmedetomidine in reducing delirium.

A typical sedation regimen in the ICU includes benzodiazepines and/or propofol. The link between benzodiazepines and delirium has been demonstrated (Zaal et al. 2015; Taipale et al. 2012), and therefore, many trials have aimed to compare these agents to dexmedetomidine. The MENDS trial was a double-blind randomized, controlled trial that found the use of dexmedetomidine to result in fewer days of delirium in medical and surgical ICU patients when compared to lorazepam (Pandharipande et al. 2007). Achieving the goal sedation level was also superior in the dexmedetomidine group, but mortality and cost did not differ when compared to the lorazepam group. A larger randomized, controlled trial SEDCOM suggested that medical and surgical ICU patients sedated with dexmedetomidine spent less time on the ventilator, experienced fewer episodes of delirium, and developed less tachycardia and hypertension than patients sedated with midazolam (Riker et al. 2009). Researchers have used these findings in medical and surgical ICUs to study the effects of dexmedetomidine specifically in cardiac surgery ICU patients.

A meta-analysis including 11 randomized, controlled or cohort studies found the use of dexmedetomidine for sedation in cardiac surgery patients’ shortened ventilator time, reduced the incidence of postoperative arrhythmias, and reduced the incidence of delirium (Lin et al. 2012). An issue with this analysis is that some of the main studies included in the analysis had poor designs and variable methods to assess for delirium. The DEXCOM study was a randomized, controlled trial which showed a decreased duration of delirium (5 vs. 2 days, (95 % CI 1.09 to 6.67); *P* = 0.0317) in patients sedated with dexmedetomidine versus morphine (Shehabi et al. 2009). The study found no difference in incidence of delirium, although the baseline delirium rate was only 15 %. Propofol was used in both study groups, and when assessing delirium, the CAM-ICU scale was used on both intubated and extubated patients. A prospective, randomized, open-label trial comparing dexmedetomidine to propofol to midazolam for sedation after cardiac surgery found an incidence of only 3 % in the dexmedetomidine group versus 50 % in both the propofol and midazolam groups (Maldonado et al. 2009). The large reduction in incidence of delirium is unprecedented as no other study has shown such a drastic reduction. A retrospective cohort consisting of data collected from 250 hospitals found that patients receiving dexmedetomidine/propofol/midazolam had a decreased incidence of delirium compared to the propofol/midazolam group, but the method for assessing delirium was not described (Dasta et al. 2006). Also, the primary endpoint of this study was cost, which was also reduced in the dexmedetomidine/propofol/midazolam group.

More recent studies continue to support the use of dexmedetomidine after cardiac surgery for reduction of delirium. When comparing dexmedetomidine to remifentanil for sedation after cardiac surgery, a reduction in the incidence of delirium was shown in the dexmedetomidine group (8.96 vs. 22.67 %; *P* < 0.05) (Park et al. 2014). The study found no differences between the two groups with respect to length of stay in the ICU, time to extubation, hospital length of stay, and other postoperative complications. Another recent single-blinded, prospective randomized trial found that when compared to propofol, the use of dexmedetomidine for sedation after cardiac surgery resulted in reduced incidence (odds ratio (OR) 0.46 (95 % CI 0.23 to 0.92); *P* = 0.028), delayed onset, and also shorter duration of delirium (Djaiani et al. 2015). A retrospective cohort investigating outcomes after CABG found a decreased mortality in patients receiving dexmedetomidine perioperatively (Ji et al. 2014). A reduction in the risk of delirium in patients receiving dexmedetomidine (adjusted OR, 0.431 (95 % CI 0.265 to 0.701); *P* = 0.0007) was also reported when compared to patients who did not receive dexmedetomidine. A larger retrospective study including CABG and CABG plus valve surgery patients also found a reduction in delirium (5.46 vs. 7.42 %; adjusted OR, 0.53 (95 % CI 0.37 to 0.75); *P* = 0.003) and decreased 1-year mortality (3.17 vs. 7.95 %; adjusted OR, 0.47 (95 % CI 0.312 to 0.701); *P* = 0.0002) in patients receiving dexmedetomidine (Ji et al. 2013).

Other interventions are also being studied. The use of high-dose dexamethasone (1 mg/kg) in cardiac surgery patients failed to show a difference in the incidence or duration of delirium (Sauer et al. 2014). The theory that the inflammatory response from surgery and cardiopulmonary bypass contributes to delirium drove the hypothesis of this study. The use of intraoperative Bispectral Index (BIS)-guided and end-tidal anesthetic concentration-guided depth of anesthesia protocols is another direction investigators have taken (Whitlock et al. 2014). No difference was shown with either of these monitors; however, low average volatile anesthetic dose was found to be a predictor of delirium. This may be reflective of patients with poorer health and could potentially assist with screening for patients at higher risk for delirium.

A systematic review and meta-analysis supported the use of dexmedetomidine, antipsychotics, and multicomponent interventions for strategies to reduce postoperative delirium based on the review of 38 randomized controlled trials but did state that one of the greatest obstacles in evaluating delirium studies is the lack of continuity in evaluation of delirium (Zhang et al. 2013). This is a major issue when clinicians are trying to extrapolate these findings and utilize them in clinical practice. When the majority of the randomized clinical trials on postoperative delirium show vast inconsistencies in the method chosen to evaluate delirium, the evidence may seem insufficient and unreliable by intensivists. And even though evidence suggests benefits of using certain interventions to reduce postoperative delirium after cardiac surgery, more studies are necessary which aim to universalize the assessment of delirium.

### Future studies

Studies on this topic are not lacking in quantity but more so in quality. Establishing a reliable, convenient, and consistent method for diagnosing delirium is a must for researchers in this field. More recent studies utilize multiple assessment tools, but until a universal screening method for delirium is accepted, it will be difficult for studies to change clinical practice with their results.

Projects involving biomarkers and their role in delirium after cardiac surgery are limitless. Aiming to target biomarkers shown to have a correlation with delirium in non-cardiac surgery patients should be investigated in cardiac surgery. Biomarkers linked to the mechanisms of delirium should also be characterized potentially through targeting biomarkers of the neuroinflammatory pathway of endothelial dysfunction, BBB disruption, and neuronal injury. This may lead to identifying patients at higher risk, preemptively treating those patients, and further defining the severity of disease in patients with delirium. Finding ways to effectively treat cerebral oxygen desaturations during CPB may also reduce postoperative delirium.

Modifications to medications administered before, during, and after surgery should continue to be investigated. The avoidance of medications such as benzodiazepines and opiates by using other sedatives and analgesics has proven to decrease delirium. Considering the flaws in studies using dexmedetomidine in the ICU, more research is necessary with this medication to determine the optimal dosing and timing of its use and solidify the evidence that it decreases delirium after cardiac surgery. The use of IVA may limit opiate consumption and play a role in decreasing delirium in these patients. An ongoing trial investigating the effects of both dexmedetomidine and IVA on delirium following cardiac surgery may provide sufficient evidence to support the use of these medications in the prevention of postoperative delirium (NCT02546765). Other analgesics such as gabapentin and pregabalin given preoperatively should also be considered.

## Conclusions

Delirium is an ongoing issue in cardiac surgery patients. Many risk factors are known, and some can be modified perioperatively. The complications associated with postoperative delirium lead to increased morbidity and mortality as well as higher healthcare costs. Several interventions in the ICU after cardiac surgery have been investigated, but the results have yet to significantly change clinical practice. The role of biomarkers in postoperative delirium after cardiac surgery is promising but not yet fully elucidated. More research and randomized clinical trials in this field is both warranted and necessary given the negative outcomes associated with delirium.

## Abbreviations

BBB: blood brain barrier
BIS: Bispectral Index
CABG: coronary artery bypass grafting
CAM-ICU: confusion assessment method for the ICU
CPB: cardiopulmonary bypass
DDS: Delirium Detection Score
DSM-V: Diagnostic and Statistical Manual, 5th edition
ICU: intensive care unit
IGF-1: insulin-like growth factor-1
IV: intravenous
IVA: intravenous acetaminophen
MMSE: Mini-Mental State Examination
NSAIDs: nonsteroidal anti-inflammatory drugs
Nu-DESC: Nursing Delirium Screening Scale
UCHL1: ubiquitin C-terminal hydrolase L1
